# Peripheral and central auditory dysfunction in tinnitus with clinically normal hearing

**DOI:** 10.1038/s41598-026-36096-y

**Published:** 2026-01-23

**Authors:** Sphoorthy Suresh, Archana Gundmi, Sanjana Madhukesh, Hari Prakash Palaniswamy

**Affiliations:** Department of Speech and Hearing, Manipal College of Health Professions (MCHP), Manipal Academy of Higher Education (MAHE), Manipal, Karnataka India

**Keywords:** Auditory health, Cochlear synaptopathy, High-frequency audiometry, Hidden hearing loss, Temporal processing deficits, Tinnitus, Diseases, Health care, Medical research, Neurology, Neuroscience

## Abstract

Tinnitus is the perception of ringing in the ear without an external source and occurs even in individuals with clinically normal hearing thresholds. This suggests there may be an undetected auditory dysfunction present. Two main mechanisms that could explain this phenomenon are ultra-high frequency hearing loss (> 8 kHz) and cochlear synaptopathy, which can disrupt both peripheral and central auditory processing. While ultrahigh frequency (UHF) audiometry is increasingly used in tinnitus assessment, the study aims to bridge the gap by systematic integration of measures of central auditory processing (TFS-AF and AMD) with clinically valid peripheral assessment (UHF) to characterise both peripheral and central deficits in tinnitus with normal hearing. The case-control study compared 28 patients with tinnitus and 28 age- and gender-matched controls. The individuals with tinnitus were assessed for tinnitus severity using the Tinnitus Functional Index (TFI). The measures included UHF audiometry (9–20 kHz), TFS-AF, and AMD tasks. The diagnostic accuracy was evaluated using the Receiver Operating Characteristic (ROC) curve, along with regression and Bland-Altman analyses. Tinnitus individuals differed across all measures (TFS-AF, AMD, and UHF) from the control group. indicating a disrupted temporal processing ability. Bland-Altman plots revealed systematic biases: TFS-AF values were consistently higher than UHF thresholds, while AMD scores were lower. UHF audiometry demonstrated superior diagnostic accuracy in classifying individuals with normal hearing who have tinnitus (96.43%). In terms of clinical utility, UHF may offer higher sensitivity in detecting tinnitus in individuals with normal hearing. Systemic biases between measures suggest the presence of independent peripheral/central deficits in individuals with normal hearing who have tinnitus.

**Supplementary Information:**

The online version contains supplementary material available at 10.1038/s41598-026-36096-y.

## Introduction

Tinnitus, a condition defined by the perception of sound, such as ringing or buzzing in the ears, occurs without an external auditory stimulus. It is primarily associated with cochlear hearing loss, yet intriguing evidence suggests that a significant proportion of tinnitus cases—approximately 10–15%—manifest in individuals who present with clinically normal hearing thresholds, consisting of the frequency range of 0.25 to 8 kHz^1^. Furthermore, tinnitus can have non-auditory origins, such as from somatosensory systems (somatic tinnitus), which is particularly common in individuals with normal hearing. This paradox indicates that conventional audiometric evaluations may not fully capture the complexities of tinnitus, necessitating a deeper investigation into alternative etiological factors.

Two predominant hypotheses have emerged to explain the occurrence of tinnitus in individuals with normal hearing thresholds. The first hypothesis posits the presence of subtle cochlear damage within the ultra-high frequency (UHF) region, defined as frequencies greater than 8 kHz^2–7^. The second hypothesis introduces the concept of hidden hearing loss (cochlear synaptopathy), which suggests that damage may specifically affect low-spontaneous-rate (low-SR) auditory nerve fibres^1,8,9^. The interaction between UHF hearing loss and synaptopathy is a subject of ongoing debate within the auditory research community. UHF hearing deficits are indicative of dysfunction in cochlear outer hair cells, which play a crucial role in amplifying sound, while synaptopathy reflects a degradation in the synapses connecting inner hair cells to auditory nerve fibres^1,10^.

Assessing synaptopathy in humans is challenging and often relies on indirect behavioural measures. TFS sensitivity, the ability to use fine-grained timing information in sounds, is thought to be dependent on the precise neural phase locking of the auditory nerve, which can be degraded by synaptopathy^11^. Therefore, deficits in TFS processing may serve as functional markers of underlying synaptic damage. Similarly, the AMD, which assesses the ability to detect spectral frequency depth of a sound, is also considered a sensitive measure of central auditory processing that may be affected by deafferentation and subsequent neural changes^12^.

Animal models have provided compelling evidence that both UHF hearing thresholds and low-SR fibres can be adversely affected by acoustic overexposure, further complicating the understanding of tinnitus’s underlying mechanisms^13^. However, research directly linking these phenomena in humans remains sparse. While UHF hearing loss can be quantified using extended audiometric testing, the assessment of synaptopathy requires indirect measurement approaches, such as temporal processing tasks (e.g., gap detection test, temporal modulation transfer function, temporal fine structure adaptive) and the amplitude-modulation detection task^8,14^. Notably, current diagnostic methodologies often fail to adequately address potential subclinical auditory deficits in individuals with normal hearing who experience tinnitus. The TFS-AF task is particularly sensitive to deficits in neural phase-locking, a fundamental process for encoding temporal fine structure that is thought to be degraded in cochlear synaptopathy^15^. Unlike other temporal tasks that can be influenced by loudness cues, it is designed to isolate the fidelity of fine-timing information. Complementing this, the AMD task assesses the auditory system’s ability to resolve a detail (i.e., spectral ripples), a function of central auditory processing that can be compromised following deafferentation and subsequent neural reorganization^12^.

Thus, both UHF audiometry and temporal processing assessments could play pivotal roles in distinguishing between cochlear and neural dysfunctions that contribute to tinnitus. However, three critical gaps in the literature must be recognized: (1) while UHF audiometry is now established in tinnitus clinical practice, there remains a lack of integrative studies that systematically combine UHF thresholds with validated central processing measures (TFS-AF/AMD) to delineate peripheral versus central contributions to tinnitus; (2) there is an absence of research investigating the comparative diagnostic utility of these combined measures for the accurate identification of tinnitus; and (3) there is an unexplored correlation between UHF thresholds and temporal processing capabilities that may yield insights into shared or independent pathophysiological mechanisms.

While the use of UHF audiometry has been established in many clinics, its integration with central processing measures, such as TFS-AF and AMD, is not. This study systematically compares these central and peripheral measures in a single tinnitus cohort to examine diagnostic differentiation and clinical utility. We hypothesise that individuals with tinnitus exhibit elevated UHF thresholds and poorer TFS-AF and AMD performance compared to controls. We further hypothesize that these measures are independent, reflecting a dual-mechanism model of tinnitus pathophysiology.

## Methods

This study included 56 participants (28 with tinnitus and 28 controls), aged 18–43 years. The tinnitus group (12 males, 16 females) reported chronic subjective tinnitus lasting at least 3 months. All participants had normal hearing thresholds (< 20 dB HL, 250 Hz–8 kHz). Controls were age- and gender-matched. Both groups met the inclusion criterion of having normal hearing thresholds (< 20 dB HL from 250 Hz to 8 kHz). Participants with known neurological, psychological, or cognitive disorders, recent tinnitus interventions, or ototoxic drug history were excluded. Participants were not screened for somatic or somatosensory tinnitus. The overall study was carried out in accordance with standard guidelines and regulations. The study protocol was reviewed and approved by the Institutional Ethics Committee (IEC2: 417/2023). Written informed consent was obtained from all participants prior to their inclusion in the study. The study does not include any identifying information or images of participants. All data are anonymised.

### Test environment and instrumentation

The experimental assessments were conducted within a carefully controlled double-walled sound-treated booth that adhered to the standards set forth by the American National Standards Institute (ANSI S3.1). This environment was instrumental in ensuring that ambient noise levels were maintained below 35 dBA SPL, minimizing external influences that could compromise the integrity of the auditory testing procedures.

For audiometry, the Madsen Astera2 audiometer (manufactured by GN Otometrics in Denmark) was utilized alongside Sennheiser supra-aural headphones, which provided high-fidelity sound presentation essential for accurate threshold determination. The TFS-AF testing was conducted using custom MATLAB scripts (MathWorks, USA) and utilizing Audio-Technica ATH-M20X headphones. The AMD testing was conducted using the PsychoPy software platform. Rigorous SPL calibration of all equipment was performed using a Class I sound level meter and ear couplers prior to testing, ensuring precise measurement and the reliability of results.

### Stimuli and procedures

#### Ultra-high frequency (UHF)audiometry

Ultrahigh-frequency (UHF) audiometry was employed to capture pure-tone thresholds from 9 to 20 kHz for both ears. The Hughson‒Westlake method, which incorporates an ascending‒descending approach, was utilized for these assessments with a step size of 5 dB. This methodology aligns with established protocols reported in tinnitus-related literature, particularly in studies conducted by Shim et al^16^. and Song et al^6^.. Intermediate frequencies were chosen on the basis of clinical evidence highlighting their relevance to identifying cochlear damage associated with tinnitus. Notably, no masking techniques were employed during UHFA testing; the elevated interaural attenuation of UHF tones presented through supra-aural headphones allowed for accurate threshold assessment without interference from the other ear. Data collection included recording thresholds at each specified frequency, culminating in the calculation of a UHF average, thereby providing a comprehensive overview of high-frequency hearing sensitivity.

#### Temporal fine structure adaptive frequency (TFS-AF)

The temporal fine structure sensitivity assessment was conducted using the TFS-AF paradigm developed by Füllgrabe and Moore^15^. Participants engaged in a two-interval, forced-choice lateralization task designed to examine their ability to discern interaural phase difference (IPD) shifts across two intervals consisting of four 400-ms tones separated by 100-ms gaps. In this setup, one interval presented tones with a standard 0° IPD, while the other featured tones with phase shifts, designated as the target. Testing started at a frequency of 250 Hz, with an initial phase difference set at 180°. The task measured the highest frequency at which a participant could reliably detect an IPD shift, with the final threshold recorded in Hz. The stimulus presentation level was set to 40 dB SPL to reduce the influence of loudness cues and focus on temporal processing. An adaptive, 2-up, 1-down staircase procedure was used to adjust the tone frequency based on participant responses, aiming for 71% correct performance. Testing stopped after eight reversals, and the final threshold was calculated as the mean of the last six reversals. Participants were instructed to select the interval where tones appeared to lateralize (shift toward one ear). Practice trials (*n* = 5) ensured task understanding.

#### Amplitude modulation detection (AMD)

To assess amplitude modulation detection, participants were asked to identify amplitude modulation within narrowband noise using a three-alternative, forced-choice detection method. The experimental stimulus consisted of a 5 kHz tone modulated at 19 Hz, referred to as the target, embedded within a 5 kHz narrowband noise (NBN) masker presented at a consistent level of 40 dB SPL. The modulation depth was initially set at 6 dB (representing 50%) and was adapted throughout the testing using the Parameter Estimation by Sequential Testing (PEST) method. Each testing interval presented three sequential tones, each lasting one second, interspersed with one-second silent gaps; only one of the tones was modulated. Participants were instructed to indicate which of the three tones exhibited modulation or vibratory changes, using labelled response keys (A, S, D) to ensure accuracy in their responses. The primary outcome was the amplitude modulation detection threshold (in dB), representing the minimum depth of modulation the participant could reliably detect. This task assesses the auditory system’s ability to resolve the amplitude details of a sound, which can be impaired following deafferentation and subsequent reorganization of the central auditory system. The 5 kHz carrier was selected to target the cochlear region most vulnerable to synaptopathy^8^and align with prior tinnitus studies^17^.

### Administration

Initially, participants in the tinnitus group completed a comprehensive history questionnaire that aimed to elucidate critical details regarding the onset, duration, type, and laterality of their tinnitus, as well as the Tinnitus Functional Index (TFI) to quantify the impact of tinnitus on their daily lives. Following these assessments, individuals with tinnitus underwent tinnitus pitch and loudness matching trials to characterize tinnitus perception. The main session involved a standardized testing protocol for both the tinnitus and control groups. Participants completed pure-tone audiometry and UHF audiometry assessments to ascertain hearing thresholds with precision. Subsequently, they engaged in temporal processing tests, specifically the TFS-AF and AMD tasks. To mitigate the potential influence of test fatigue and learning effects, each task was administered in a randomized order, thereby promoting the reliability of the results while ensuring a robust assessment of both auditory processing capabilities and fundamental hearing thresholds.

### Data analysis

All statistical analyses were performed using Jamovi (version 2.7.12^18^;, an open-source statistical software platform that provides an accessible interface to R. Descriptive statistics were calculated for TFS-AF, AMD, and UHFA, expressed as mean ± standard deviation (SD), to provide a clear statistical context. Independent samples t-tests were used to compare group means, enabling the identification of significant differences between the tinnitus and control groups. Furthermore, Pearson’s correlation coefficients were calculated to explore potential relationships between the various auditory measures, offering insights into the interplay between tinnitus and auditory processing abilities. Lastly, Receiver Operating Characteristic (ROC) curve analyses were conducted to evaluate the diagnostic accuracy of the employed tests, providing valuable information regarding the potential of these auditory assessments in clinical applications. This analysis is useful for determining the clinical utility of a test by quantifying its ability to distinguish between two groups (in this case, tinnitus vs. control).

Linear mixed-effects models (LMM) were estimated using the GAMLj module^19^ to examine group differences in EHFA, AMD, and TFS-AF while controlling for potential covariates. The dependent variables were the respective auditory measures, the fixed effect was Group (Tinnitus, Control), and PTA was included as a covariate. A linear regression analysis was also performed to investigate the relationship between auditory measures and TFI scores in the tinnitus group. The assumptions of normality and collinearity were checked; however, we acknowledge that the sample size of 28 is small for regression analysis.

## Results

The study compared two groups: the tinnitus group and the control group. The average age of participants in the tinnitus group was 28.25 years (SD = 7.46), while the control group had an average age of 28.29 years (SD = 7.41); however, there was no significant difference between the groups (*p* = 0.0984). The Tinnitus Group had a mean TFI score of 40.9 (SD = 14.3).

**Fig. 1:**
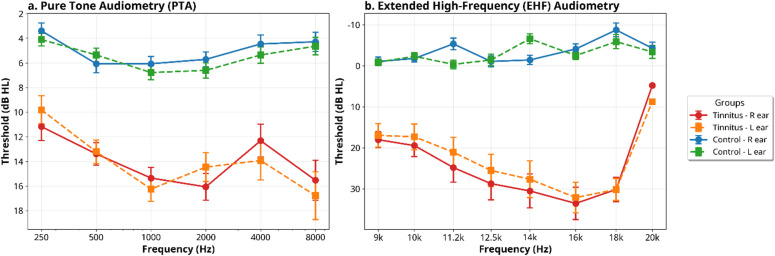
Mean hearing thresholds (dB HL) for the Tinnitus and Control groups. (a) Pure Tone Audiometry (PTA) thresholds from 250 Hz to 8000 Hz (Left). (b) Extended High-Frequency (EHF) audiometry thresholds from 9 kHz to 20 kHz (Right). Data are shown for the right (R) and left (L) ears. Error bars represent the standard error of the mean (SEM).

The Tinnitus characteristics and Pure tone average (PTA) of the participants from the tinnitus group are depicted in Table 1 and plotted in Fig. 1.

**Table 1:** Participant characteristics and their PTA (right and left ear).

Participant	Tinnitus Duration	Laterality	Type	Pitch (in Hz)	Loudness (in dB HL)	PTA-Right	PTA-Left	TFI-Total
01	5 years	Bilateral	Intermittent	6 kHz	30 dB	13.33	18.33	12.00
02	3 years	Bilateral	Continuous	2 kHz	45 dB	18.33	20.00	45.60
03	2 years	Right	Continuous	4 kHz	40 dB	16.67	13.33	47.20
04	3 months	Left	Intermittent	750 Hz	35 dB	16.67	13.33	29.60
05	2 years	Left	Intermittent	1.5 kHz	50 dB	13.33	18.33	37.60
06	6 months	Right	Intermittent	2 kHz	55 dB	6.67	6.67	33.60
07	4 months	Right	Intermittent	CND	CND	16.67	13.33	42.40
08	3 years	Left	Continuous	1 kHz	60 dB	11.67	8.33	80.00
09	1 year	Right	Intermittent	2 kHz	25 dB	11.67	11.67	22.00
10	6 months	Left	Intermittent	3 kHz	30 dB	16.67	16.67	60.00
11	1 year	Left	Continuous	4 kHz	40 dB	16.67	18.33	50.80
12	4 years	Left	Intermittent	2.5 kHz	20 dB	16.67	16.67	21.60
13	5 months	Right	Continuous	1 kHz	50 dB	13.33	11.67	59.20
14	1 year	Left	Intermittent	CND	CND	15.00	18.33	40.80
15	2 years	Left	Continuous	750 Hz	50 dB	16.67	18.33	61.20
16	1 year	Right	Intermittent	1.5 kHz	40 dB	16.67	13.33	33.60
17	2 years	Right	Continuous	2 kHz	35 dB	18.33	16.67	64.80
18	1 year	Right	Intermittent	CND	CND	16.67	13.33	34.00
19	6 months	Right	Intermittent	6 kHz	30 dB	10.00	8.33	34.40
20	2 years	Left	Intermittent	3 kHz	25 dB	13.33	13.33	58.40
21	4 years	Left	Intermittent	2 kHz	30 dB	13.33	18.33	34.00
22	7 months	Bilateral	Continuous	6 kHz	35 dB	15.00	13.33	49.60
23	4 years	Bilateral	Continuous	5 kHz	20 dB	5.00	8.33	28.00
24	2 months	Bilateral	Intermittent	3 kHz	25 dB	6.67	6.67	22.00
25	1 year	Right	Intermittent	1.5 kHz	45 dB	11.67	13.33	28.80
26	8 months	Bilateral	Continuous	6 kHz	30 dB	23.33	23.33	45.60
27	1 year	Bilateral	Intermittent	3 kHz	50 dB	21.67	23.33	44.00
28	7 months	Right	Continuous	2 kHz	55 dB	21.67	8.33	56.40

Note: CND: Could not be done, PTA – Pure Tone Average, TFI – Tinnitus Functional Index, dB HL – Decibel Hearing Level.

The means of TFS-AF, AMD, and UHFA for both the right and left ear, along with their standard deviation for both the tinnitus and control groups, are represented in Table 2.

**Table 2:** Mean and standard deviation, along with t-test statistics for both the tinnitus and control groups.

Variables	Tinnitus Group		Control Group		Statistics (t)	df	*p*	Mean diff.
	Mean	SD	Mean	SD				
PTA-R (dB HL)	14.9	4.57	5.95	2.66	−8.99	54.0	0.001	−8.99
PTA-L (dB HL)	14.6	4.93	6.25	2.38	−8.10	54.0	0.001	−8.39
UHFA-R (dB HL)	23.77	14.44	1.26	2.62	9.71	54.0	0.001	27.25
UHFA-L (dB HL)	22.46	15.62	1.56	1.80	8.39	54.0	0.001	25.38
TFS-AF (in Hz)	894.35	358.79	1165.94	234.60	−3.35	54.0	0.001	−271.59
AMD (in dB)	−11.96	3.38	−16.35	1.59	6.22	54.0	0.001	4.39

Note: PTA-R: Pure tone average for right ear, PTA-L: Pure tone average for left ear, TFS-AF: Temporal Fine Structure-Adaptive Frequency, AMD: Amplitude Modulation Detection, UHFA-R: Ultra-High Frequency Audiometry Right, UHFA-L: Ultra-High Frequency Audiometry -Left.

The analysis of UHFA using a linear mixed-effects model (LMM) revealed a significant group effect, indicating that individuals with tinnitus exhibited higher UHFA values compared to the control group, even after adjusting for pure-tone average (PTA) scores (β = 24.3, SE = 3.1, t = 7.84, *p* < 0.001). The inclusion of PTA as a covariate was insignificant (*p* = 0.12), confirming that UHFA differences are independent of routine hearing thresholds. The analysis showed no significant difference in UHFA between the left and right ears (*p* = 0.34). For the AMD thresholds, the LMM analysis again indicated a significant group effect, with the tinnitus group showing less negative AMD scores than the control group (β = 4.2, SE = 0.9, t = 4.67, *p* < 0.001). Similar to the UHFA analysis, the PTA covariate was insignificant (*p* = 0.21). Further, analysis of temporal fine structure adaptive frequency (TFS-AF) indicated that individuals with tinnitus had poorer TFS-AF scores compared to the control group (β = −280.5, SE = 85.2, t = −3.29, *p* = 0.002). These results suggest that PTA differences do not explain temporal deficits in tinnitus patients.

Pearson’s correlation coefficient was utilized to investigate the relation between the tests, and the results are presented in Table 3. The correlation analysis revealed no statistically significant relation between variables, indicating a lack of influence among TFS-AF, AMD, and UHFA. However, UHFA-R and UHFA-L were correlated with TFI-total scores. Furthermore, a significant negative correlation was found between the duration of tinnitus and AMD (Table 3).

**Table 3:** The correlation matrix explains the relationship between the variables measured in the study.

		TFS-AF	AMD	UHFA-*R*	UHFA-L	TFI-total	Duration of Tinnitus
TFS-AF	Pearson’s r	—					
	df	—					
	p-value	—					
AMD	Pearson’s r	−0.169	—				
	df	26	—				
	p-value	0.390	—				
UHF-A_R	Pearson’s r	0.355	0.030	—			
	df	26	26	—			
	p-value	0.064	0.878	—			
UHF-A_L	Pearson’s r	0.213	−0.065	0.857	—		
	df	26	26	26	—		
	p-value	0.276	0.744	**< 0.001**	—		
TFI-TOTAL	Pearson’s r	0.068	0.259	0.457	0.458	—	
	df	26	26	26	26	—	
	p-value	0.730	0.183	**0.015**	**0.014**	—	
Duration of Tinnitus	Pearson’s r	0.017	−0.384	−0.317	−0.226	−0.177	—
	df	26	26	26	26	26	—
	p-value	0.931	**0.044**	0.100	0.248	0.367	—

Note: TFS-AF: Temporal Fine Structure-Adaptive Frequency, AMD: Amplitude Modulation Detection, UHFA-R: Ultra High Frequency Average-Right, UHFA-L: Ultra High Frequency Average-Left and TFI-total: Total Tinnitus Functional Index Scores, Significant results are presented in bold.

In tinnitus patients with normal routine hearing (*n* = 28), linear regression revealed that ultra-high-frequency thresholds (UHFA-R) significantly predicted TFI severity (β = 0.51, *p* = 0.019), accounting for 27.2% of variance. AMD showed a positive but non-significant trend (β = 1.09, *p* = 0.198), while temporal fine structure (TFA-AF) was unrelated to TFI (*p* = 0.759). Assumptions of normality (Shapiro-Wilk *p* = 0.406) and low collinearity (VIFs < 1.2) were met.

A receiver operating characteristic (ROC) curve analysis was used to compare the sensitivity and specificity of the measures TFS-AF, AMD, and UHFA-R (hereafter referred to simply as UHFA). The results are expressed in Table 4.

**Table 4:** The cut point, sensitivity, and specificity of UHFA, AMD and TFS-AF.

Scale	Cutpoint	Sensitivity(%)	Specificity(%)
UHFA	0.625	96.43%	92.86%
	1.25	96.43%	96.43%
	4.375	92.86%	96.43%
AMD	−14.56	78.57%	89.29%
	−13.97	75%	92.86%
	−13.6	75%	96.43%
TFS-AF	220.20	96.43%	0%
	1394.36	10.71%	85.71%
	1710.68	0%	96.43%

Note: UHFA - Ultra High Frequency Audiometry, AMD - Amplitude Modulation Detection, TFS-AF - Temporal Fine Structure-Adaptive Frequency.

**Fig. 2:**
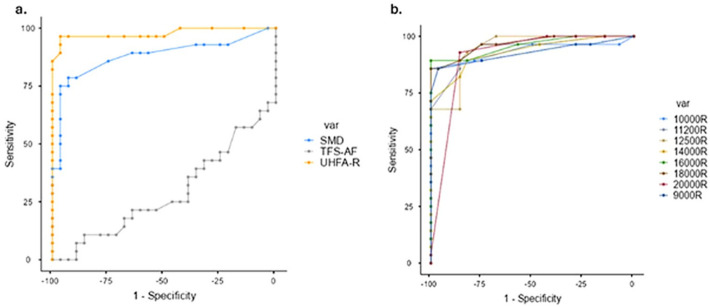
Diagnostic performance of auditory measures in individuals with tinnitus. (**a**). Receiver Operating Characteristic Curve Graph showing sensitivity and specificity of the AMD, TFS-AF, and UHFA. (**b**). Independent sensitivity of UHFs in detecting tinnitus.

ROC curve analysis was conducted to evaluate the sensitivity and specificity of several auditory measures, specifically UHFA-R, AMD, and TFS-AF, in distinguishing tinnitus patients from control subjects. The results are summarized in Table 4. Among the measures assessed, UHFA exhibited the highest diagnostic accuracy, achieving a sensitivity of 96.43% and specificity of 96.43% at a cut point of 1.25. The ROC curve for UHFA approached the ideal top-left corner, indicating its near-perfect discriminative ability, as illustrated in Fig. 1a. In comparison, AMD also demonstrated strong performance, with a sensitivity of 78.57% and specificity of 89.29% at a cut point of −14.56. Notably, the sensitivity improved to 75% with a specificity of 96.43% at higher thresholds. These findings are also detailed in Table 4; Fig. 2a.

Conversely, TFS-AF exhibited poor diagnostic utility, as evidenced by a sensitivity of 0% at a high specificity of 96.43%. This significant decrease underscores its limited effectiveness in differentiating between tinnitus patients and controls (refer to Table 4; Fig. 2a). Further analysis of individual ultra-high frequencies ranging from 9 to 20 kHz in the right ear revealed that frequencies of 10, 16, 18, and 20 kHz achieved near-perfect sensitivity across various specificity levels, as shown in Fig. 2b. This highlights the potential of specific frequencies in enhancing diagnostic accuracy for tinnitus.

### Bland-altman analysis: TFS-AF and AMD vs. UHFA

**Fig. 3:**
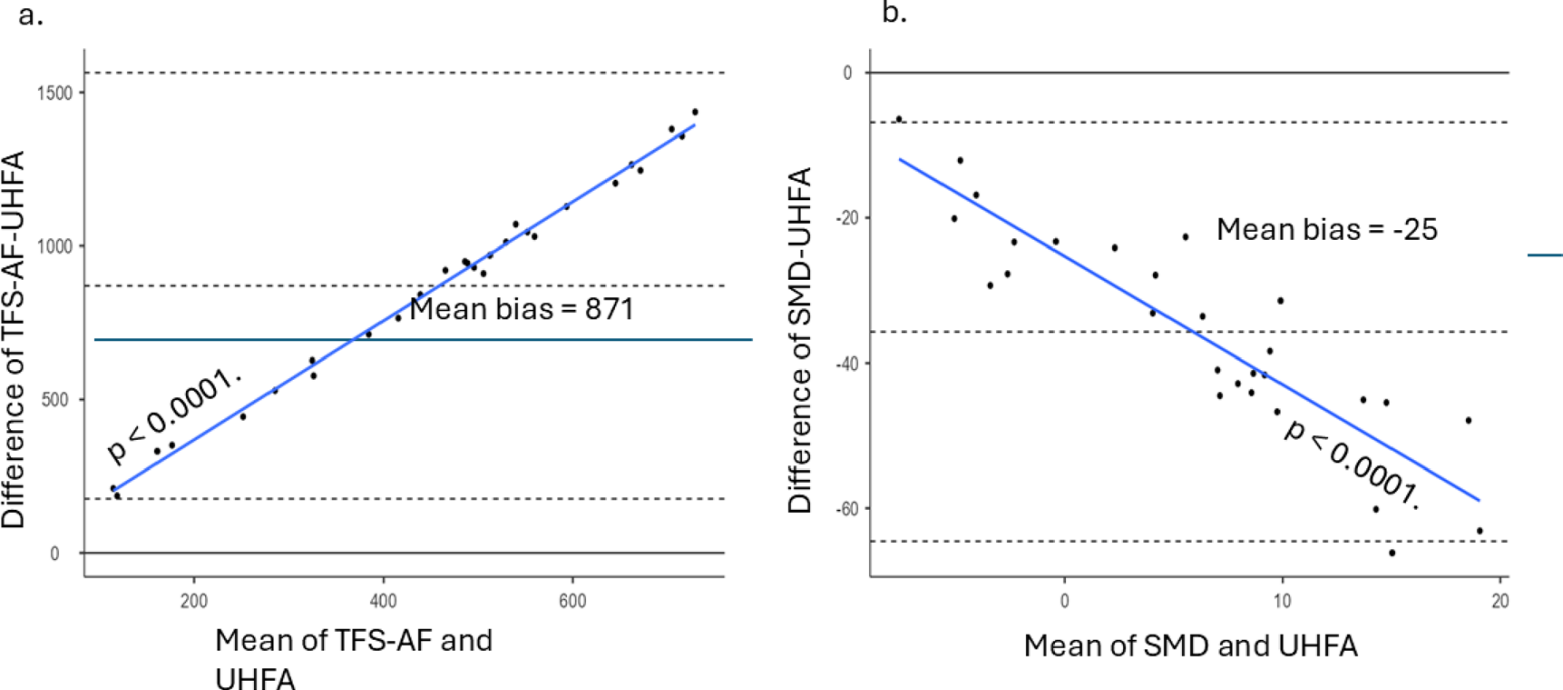
Bland-Altman analysis. (**a**). Bland-Altman plot of TFS-AF Vs. UHFA. (**b**). Bland-Altman plot of AMD Vs. UHFA.

As shown in Fig. 3a and b, the Bland-Altman analysis comparing the two measurements, TFS-AF and AMD, against the UHFA thresholds reveals significant insights into the relationship between these variables. TFS-AF vs. UHFA indicates a mean bias of 871 (95% CI: 733.3, 1008), which suggests that TFS-AF values are consistently higher than those recorded by UHFA. In contrast, the comparison of AMD with UHFA yields a mean bias of −25 (95% CI: −41.4, −30.02). This negative bias signifies that the AMD values are generally lower than those of UHFA. The limits of agreement for this pair are also notable, stretching from − 64.59 (95% CI: −74.5, −54.71) to −6.87 (95% CI: −16.7, 3.01). These results suggest that peripheral hearing loss may not be the primary cause of the observed temporal processing deficits and have potential implications for central mechanisms.

## Discussion

The findings regarding ultra-high frequency audiometry in tinnitus patients provide significant insights into the underlying auditory mechanisms associated with this condition. The observed elevation in UHF thresholds in individuals with tinnitus suggests potential cochlear damage, particularly within the basal region, even when conventional audiometric assessments remain within normal limits^20^. This aligns with the ‘hidden hearing loss’ hypothesis, which posits that cochlear synaptopathy or UHF damage may underlie tinnitus despite normal conventional thresholds^1,9^. Meta-analyses confirm UHF deficits as a hallmark in tinnitus patients^20^. This discrepancy highlights a crucial aspect of auditory health: traditional audiometric tests, which typically assess frequencies between 250 Hz and 8 kHz, may not adequately capture all auditory deficits in tinnitus patients. The “hidden hearing loss” concept emerges as a compelling framework for understanding these results^21^. It underlines the possibility that individuals with tinnitus may experience auditory challenges that are not detectable through standard testing methods, necessitating more sensitive diagnostic tools to reveal the full extent of auditory compromise.

The promising diagnostic accuracy of ultra-high frequency audiometry reinforces its potential as a valuable clinical tool for objectively assessing tinnitus. This advanced testing modality can enhance clinical practice by providing more detailed insights into patients’ auditory profiles, ultimately leading to better-targeted interventions and management strategies. As such, further exploration into the application of UHF audiometry in both research and clinical settings is warranted to unravel the complexities of tinnitus and guide effective treatment approaches.

### Temporal and amplitude modulation processing deficits

The observed differences in amplitude modulation and temporal processing abilities between tinnitus patients and control groups indicate significant abnormalities in these areas. Tinnitus patients exhibited a notable challenge in their ability to process IPDs, which is crucial for sound localization and understanding spatial auditory cues. This aspect of auditory processing is fundamental for everyday listening environments, particularly in complex acoustic scenes where distinguishing between overlapping sounds is necessary. The TFS deficit may be linked to cochlear synaptopathy, which degrades the neural phase-locking necessary for fine-grained temporal processing^11^. This finding is consistent with some previous studies^22^ but contradicts others who found no significant TFS deficits in tinnitus patients^23^. These discrepancies may be due to differences in methodology or participant characteristics, highlighting the need for further research.

Furthermore, the deficits noted in AMD capabilities among tinnitus patients suggest that these individuals may experience difficulties in distinguishing between different frequencies. This impairment may be linked to underlying neural mechanisms, potentially indicating synaptopathy, which affects auditory nerve fibers, particularly those with low SRs^8,17^. These temporal deficits mirror findings in animal models and humans with synaptopathy, where degraded neural phase locking disrupts temporal resolution^15,24^. Such damage can disrupt the normal encoding of sound frequencies, contributing to the auditory masking and distorted perception often reported by tinnitus sufferers^23^.

The implications of these findings are profound, as they suggest that tinnitus is not merely a perception issue but may also involve underlying auditory processing deficits that affect both temporal and amplitude modulation dimensions of sound. Understanding these impairments is essential for developing targeted interventions and therapies to alleviate tinnitus symptoms and improve the overall auditory experience for affected individuals. Future research should continue to investigate the neural correlates of these deficits to establish a clearer understanding of the mechanisms underlying tinnitus and its associated processing challenges.

### Dissociation between measures

The absence of significant correlations among the TFS-AF, AMD, and UHFA measures suggests that these assessments may capture different dimensions of auditory dysfunction associated with tinnitus. The findings from the Bland-Altman analysis reveal systematic differences between the measures, which further underscores the notion that peripheral damage and central processing deficits operate independently. Similar dissociations between peripheral and central measures have been reported in tinnitus cohorts, reinforcing the multi-mechanism model^2,6^. Such insights enhance the understanding of tinnitus and highlight the complexity of its underlying mechanisms.

### Relationship with tinnitus severity

The relationship between UHFA thresholds and TFI scores indicates a noteworthy connection, suggesting that the severity of peripheral damage may play a role in influencing tinnitus distress. The findings from the linear regression analysis support the notion that cochlear damage is linked to tinnitus severity. Additionally, the observed negative correlation between tinnitus duration and AMD performance implies that chronic tinnitus could lead to a decline in Amplitude processing abilities over time. These insights emphasize the complex interplay between auditory damage and the subjective experience of tinnitus^25^ highlighting the need to explore these factors further to understand tinnitus and its impact on individuals.

### Diagnostic accuracy

The findings from this study indicate a clear hierarchy in diagnostic accuracy among various measures used for assessing tinnitus. While the diagnostic value of UHF audiometry is known, this study provides the first direct, within-subject comparison of its accuracy against central processing measures. UHF audiometry demonstrated superior diagnostic accuracy (AUC ≈ 0.98) compared to AMD (Area under curve; AUC ≈ 0.85) and TFS-AF (AUC ≈ 0.50). The complementary value of AMD suggests that a comprehensive assessment may enhance clinical decision-making (Fig. 3). The superior diagnostic accuracy of UHF audiometry corroborates prior studies demonstrating its sensitivity in identifying subclinical cochlear damage^4,25^. Notably, the investigation into specific UHF revealed that^1,]^ assessing the very high frequency ranges may provide enhanced sensitivity in diagnosis.

### Theoretical implications

The independence of various measurement outcomes underscores the possibility that tinnitus may arise from multiple pathways rather than a single identifiable deficit. This is further emphasized by detecting temporal processing deficits that persist despite controlling peripheral hearing loss, indicating that central auditory dysfunction plays a significant role in tinnitus. The theoretical implications of these findings align with contemporary models that propose tinnitus arises from maladaptive neuroplastic changes in response to deafferentation^26^. This study bridges two predominant hypotheses surrounding tinnitus—those related to UHF hearing loss and synaptopathy—by presenting evidence for the independent operation of both mechanisms^8^. The connection between UHF assessment and tinnitus severity further substantiates the idea that peripheral damage can trigger a cascade of central changes that culminate in the conscious experience of tinnitus.

These results emphasise the need for a comprehensive approach to tinnitus assessment, integrating peripheral and central measures to enhance diagnostic accuracy and inform treatment strategies. These findings collectively advance the understanding complex pathophysiology underlying tinnitus in individuals with clinically normal hearing, highlighting the importance of comprehensive audiological assessment beyond conventional measures.

## Limitations

While this study provides valuable insights into the interplay between peripheral and central auditory dysfunction in tinnitus patients with normal hearing, several limitations must be acknowledged to contextualize the findings appropriately. First, the sample size (*n* = 28 per group) was relatively small. Although sufficient for the statistical analyses conducted, a larger cohort would increase the statistical power and improve the generalizability of the findings. Second, participants were not screened for somatic tinnitus, which is a potential confounder. Third, the measures used to assess central auditory processing deficits, TFS-AF and AMD, are indirect behavioral proxies for cochlear synaptopathy. While these tests are sensitive to the functional consequences of neural deafferentation, they are not direct physiological measures of synaptic integrity. Therefore, the interpretation of deficits in these tasks as direct evidence of cochlear synaptopathy should be approached with caution. Future research incorporating electrophysiological measures, such as the Wave I amplitude of the auditory brainstem response (ABR), would be beneficial for a more direct assessment of the auditory nerve condition. Fourth, this study did not include measures of loudness discomfort levels (LDLs). Given the high comorbidity between tinnitus and hyperacusis, the inclusion of LDLs would have provided valuable insights into the potential role of sound sensitivity in this cohort and its relationship with the observed behavioral measures. Additionally, the study did not analyze the effects of tinnitus laterality on the auditory measures. While the laterality was documented (Table 1), the unequal and relatively smaller sub-group sizes restricted meaningful statistical comparison. For example, the left-sided tinnitus group (*n* = 6) had insufficient power for robust statistical inference. Future research should include laterality as a between-group variable in statistical models. This would strengthen understanding how lateralized cochlear pathology is related to the outcome measures. Finally, the potential confounding effect of UHFA hearing loss on central processing measures cannot be fully ruled out. Damage to the basal, high-frequency region of the cochlea can trigger downstream neural plasticity and reorganization in the central auditory system. Consequently, it is plausible that the observed deficits in TFS-AF and AMD performance may be, at least in part, a consequence of the peripheral damage reflected in the elevated UHF thresholds rather than an entirely independent central deficit.

## Supplementary Information

Below is the link to the electronic supplementary material.

Supplementary Material 1

## Data Availability

All data generated or analysed during this study are included as supplementary Information.
